# Metabolomic Analysis of Differential Changes in Metabolites during ATP Oscillations in Chondrogenesis

**DOI:** 10.1155/2013/213972

**Published:** 2013-06-26

**Authors:** Hyuck Joon Kwon, Yoshihiro Ohmiya

**Affiliations:** ^1^Department of Physical Therapy, College of Health Science, Eulji University, Gyeonggi 461-713, Republic of Korea; ^2^National Institute of Advanced Industrial Science and Technology, Biomedical Research Institute, Tsukuba 305-8566, Japan

## Abstract

Prechondrogenic condensation is a critical step for skeletal pattern formation. Recent studies reported that ATP oscillations play an essential role in prechondrogenic condensation. However, the molecular mechanism to underlie ATP oscillations remains poorly understood. In the present study, it was investigated how changes in metabolites are implicated in ATP oscillations during chondrogenesis by using capillary electrophoresis time-of-flight mass spectrometry (CE-TOF-MS). CE-TOF-MS detected 93 cationic and 109 anionic compounds derived from known metabolic pathways. 15 cationic and 18 anionic compounds revealed significant change between peak and trough of ATP oscillations. These results implicate that glycolysis, mitochondrial respiration and uronic acid pathway oscillate in phase with ATP oscillations, while PPRP and nucleotides synthesis pathways oscillate in antiphase with ATP oscillations. This suggests that the ATP-producing glycolysis and mitochondrial respiration oscillate in antiphase with the ATP-consuming PPRP/nucleotide synthesis pathway during chondrogenesis.

## 1. Introduction

Skeletal development in vertebrate limb begins with chondrogenesis in which prechondrogenic cells condense and then differentiate into chondrocytes to form a variety of precisely shaped cartilage elements that are ultimately replaced by bone tissues through endochondral ossification [1]. This indicates that the prechondrogenic condensation is a critical step for skeletal pattern formation in limb development. We recently found that ATP oscillations play a key role in prechondrogenic condensation during chondrogenesis [2–4]. It was demonstrated that extracellular ATP and cAMP/PKA signaling mediate ATP oscillations during chondrogenesis [5]. However, how metabolic pathways are liked with ATP oscillations remains poorly understood. 

Metabolites are the main effectors of phenotype and functional entities in the cell [6]. Thus, metabolomics, which is defined as the measurement of the level of all intracellular metabolites, is a powerful tool for gaining insight into cellular functions. Indeed, metabolomic analyses are being increasingly performed to clarify biochemical mechanisms to underlie various physiological processes [7–9]. Therefore, comprehensive profiles of differential metabolite changes during metabolic oscillations can reveal the connection of biochemical networks during metabolic oscillations and thus provide a system-level understanding of metabolic oscillations during chondrogenesis. 

Although most metabolic analyses have been performed with gas chromatography mass spectrometry (GC-MS) [10], GC-MS is limited by the need for multiple deprivation procedures for each chemical moiety and the fact that nonvolatile, thermolabile, and highly polar compounds are difficult to be determined. However, capillary electrophoresis mass spectrometry (CE-MS) in which metabolites are separated by CE based on charge and size and then selectively detected using MS has the major advantages in extremely high resolution, high throughput, and ability to simultaneously quantify all charged low-molecular weight compounds [11–13]. Thus, CE-MS has emerged as a useful tool for analyzing polar and charged molecules [13–15]. 

In the present work, capillary electrophoresis time-of-flight mass spectrometry (CE-TOF-MS) was applied to the metabolome profiling of differential metabolite during ATP oscillations in chondrogenesis. This CE-TOF-MS method covered a wide (50–1000) *m*/*z* range. This system determined 93 cationic and 109 anionic compounds derived from known metabolic pathways in prechondrogenic cell line ATDC5 cells and revealed significant change of 15 cationic and 18 anionic compounds between peak and trough of ATP oscillations. This study provides information about how metabolic pathways are involved in ATP oscillations during chondrogenesis. 

## 2. Materials and Methods

### 2.1. Cell Line

The ATDC5 cell line was obtained from the RIKEN cell bank (Tsukuba). The cells were cultured in maintenance medium consisting of a 1 : 1 mixture of Dulbecco's modified Eagle's medium and Ham's F-12 medium (DMEM-F12) (Invitrogen) supplemented with 5% fetal bovine serum, 10 mg/mL human transferrin (Roche Molecular Biochemicals), and 3 × 10^−8^ M sodium selenite (Sigma-Aldrich) in polystyrene dishes at 37°C under 5% CO_2_. For chondrogenic induction, when ATDC5 cells maintained in the maintenance medium reached confluency, the medium was replaced with the chondrogenic medium supplemented with 10 *μ*g/mL insulin (Sigma-Aldrich).

### 2.2. Bioluminescence Monitoring Experiments

Bioluminescence reporter constructed by fusing the human actin promoter to a* Phrixothrix hirtus *luciferase gene (Toyobo) was inserted into retrovirus vector (Clontech). ATDC5 cells were transfected using retrovirus infection and then were selected by puromycin. The cells transfected stably with P_ACTIN_-PxRe were seeded in 35 mm dishes. After replacing the medium by a recording medium (DMEM/F12 with 5% FBS, 0.1 mM luciferin (Wako), and 50 mM HEPES-NaOH, pH = 7.0) (time = 0 h), bioluminescence intensity was continuously measured at 37°C in air using a dish-type luminescence, Kronos (ATTO) for 1 min at 30 min intervals. For examining the effect of chemical compounds on ATP oscillations, the cells were treated with 0.1 mM iodoacetate (Sigma-Aldrich) or 5 mM cyanide (Sigma-Aldrich) about 24 hours after P_ACTIN_-PxRe oscillations initiated.

### 2.3. Sample Preparation for CE-TOF-MS Analysis

ATDC5 cells cultured to 1 × 10^6^ cells needed pretreatment. The culture medium was removed from the plates, and 10 mL of 5% mannitol solution was added to wash the cells. After 2 mL of 5% mannitol solution was added to wash the cells, 1,300 *μ*L of methanol containing the internal standards was added. Cells were scraped from the plate and then cell lysate (~1,000 *μ*L) was used in the analysis. Then, 400 *μ*L of Milli-Q water and 1,000 *μ*L of chloroform were added to the samples, thoroughly mixed, and then centrifuged for 5 min at 2,300 g and 4°C. The 750 *μ*L of upper aqueous layer was centrifugally filtered through a Millipore 5 kDa cutoff filter to remove proteins. The filtrate was lyophilized and suspended in 25 *μ*L of Milli-Q water. We prepared three samples at peak and trough of P_ACTIN_-PxRe oscillations, respectively, and used each sample for independent CE-TOF-MS analysis.

### 2.4. Instrumentation for CE-TOF-MS Analysis

The measurement of extracted metabolites was performed by using a capillary electrophoresis- (CE-) connected ESI-TOF-MS system with electrophoresis buffer (Solution ID H3302-1021, Human Metabolome Technologies Inc., Tsuruoka, Japan). CE-TOF-MS was carried out using an Agilent CE Capillary Electrophoresis System equipped with an Agilent 6210 Time of Flight mass spectrometer, Agilent 1100 isocratic HPLC pump, Agilent G1603A CE-MS adapter kit, and Agilent G1607A CE-ESI-MS sprayer kit (Agilent Technologies, Waldbronn, Germany). The system was controlled by Agilent G2201AA ChemStation software version B.03.01 for CE (Agilent Technologies, Waldbronn, Germany). 

### 2.5. Experimental Conditions for CE-TOF-MS Analysis

For cationic metabolites, capillary electrophoreses were performed using a fused silica capillary. The electrolyte was 1 M formic acid. Methanol-water (50% v/v) containing 0.5 mM reserpine (the lock mass for exact mass measurements) was delivered as the sheath liquid. For anionic metabolites, separations were performed using a fused silica capillary. The electrolyte was 50 mM ammonium acetate (pH 8.5). The sheath liquid for anionic metabolites was used as the electrolyte. The capillary was pretreated with preconditioning buffer including 25 mM ammonium acetate and 75 mM sodium phosphate at pH 8.5. Pressure of 5 mbar was applied to inlet capillary during run to reduce the analysis time. For all analytical modes, inner diameter and total length of capillary are 50 mm and 80 cm, respectively. The applied voltage was set at +30 kV and −30 kV for cation and anion mode, respectively. Electrospray ionization TOF-MS was operated in the positive ion mode (4 kV), the negative ion mode (3.5 kV) for cationic metabolites, anionic metabolites, and nucleotides, respectively. A flow rate of heated dry nitrogen gas (heater temperature 300°C) was maintained at 5 psig. Exact mass data were acquired over a 50–1000 *m*/*z* range. Signal peaks corresponding to isotopomers, adduct ions, and other product ions of known metabolites were excluded, all signal peaks potentially corresponding to authentic compounds were extracted, and then their migration time (MT) was normalized using those of the internal standards. Thereafter, the alignment of peaks was performed according to the *m*/*z* values and normalized MT values. Finally, peak areas were normalized against those of the internal standards, MetSul and CSA for cations and anions, respectively. The resultant relative area values were further normalized by sample amount. Annotation tables were produced from CE-ESI-TOF-MS measurement of standard compounds and were aligned with the datasets according to similar *m*/*z* values and normalized MT values. 

## 3. Results and Discussion 

### 3.1. Relationship of Glycolysis and Mitochondrial Respiration with ATP Oscillations

**Figure 1:**
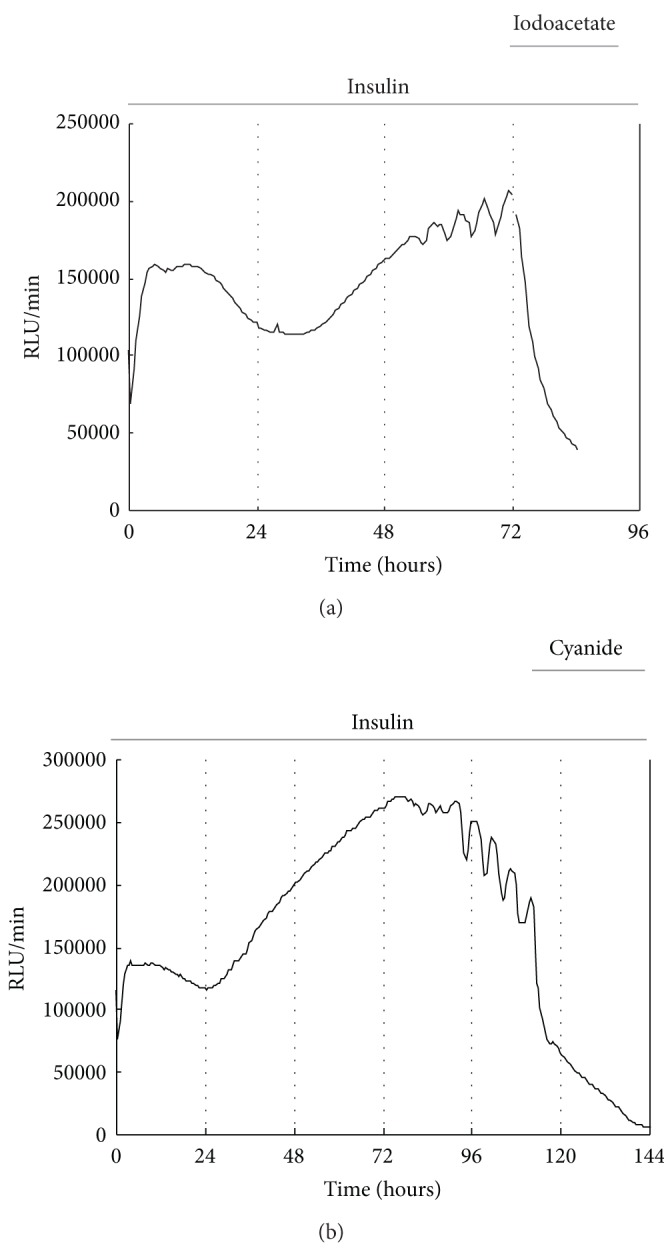
ATP oscillations are linked with both glycolysis and mitochondrial respiration. Either a glycolysis inhibitor iodoacetate or an inhibitor of mitochondrial respiration cyanide eliminates insulin-induced P_ACTIN_-PxRe oscillations in ATDC5 cells. Bioluminescence monitoring of P_ACTIN_-PxRe activity in ATDC5 cells was performed after chondrogenic induction with the chondrogenic medium (time = 0 h). About 24 hours after P_ACTIN_-PxRe oscillations were initiated, ATDC5 cells were treated with iodoacetate or cyanide.

In this study, insulin which stimulates chondrogenesis of ATDC5 cells [16] was used in induction of ATP oscillations during chondrogenesis. Intracellular ATP level of ATDC5 cells was monitored in real time by transfection of ATP-dependent *Phrixothrix hirtus *luciferase (PxRe) reporter gene fused to a constitutive ACTIN promoter (P_ACTIN_-PxRe) into the cells. Consistent with the previous studies [2–4], we found that ATP oscillations were generated 2–4 d after chondrogenic induction (Figure 1). Since ATP is produced mainly by glycolysis and mitochondrial respiration, we examined whether ATP oscillations depend on glycolysis or mitochondrial respiration. It was shown that both a glycolysis inhibitor iodoacetate and a mitochondrial respiration inhibitor cyanide suppressed insulin-induced ATP oscillations (Figure 1). This result indicates that ATP oscillations in chondrogenesis depend on both glycolysis and mitochondrial respiration. 

### 3.2. Heatmap for Differential Changes of Metabolites between Peak and Trough of ATP Oscillations

**Figure 2:**
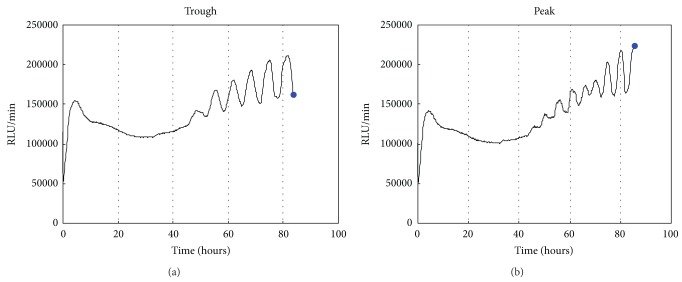
Blue dots represent trough and peak of P_ACTIN_-PxRe oscillations in which metabolites were extracted from ATDC5 cells. Bioluminescence monitoring of P_ACTIN_-PxRe activities was performed after chondrogenic induction (time = 0 h).

**Figure 3:**
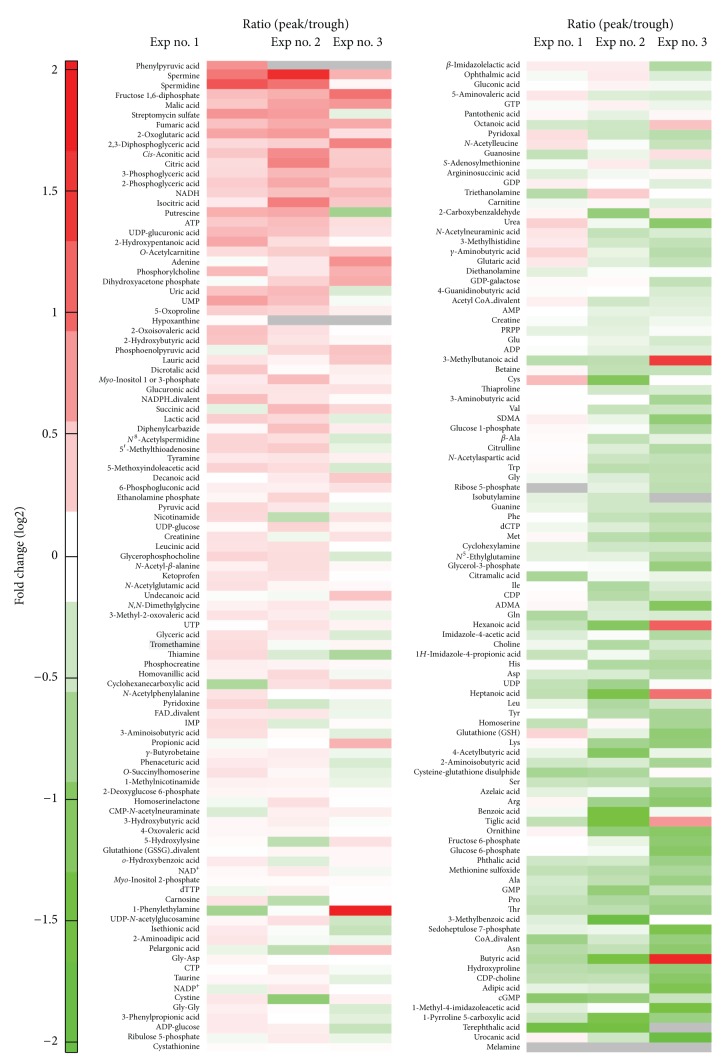
The result of the metabolite analysis is illustrated in a heatmap, which represents the fold change of metabolite concentrations in peaks of P_ACTIN_-PxRe oscillations compared with those in troughs of P_ACTIN_-PxRe oscillations. Data are from three independent experiments (Exp nos. 1–3). Gray means the case in which the metabolite was undetected in peak or trough of P_ACTIN_-PxRe oscillations.

To elucidate the metabolic pathways to generate ATP oscillations during chondrogenesis, the differential changes in the metabolites between peak and trough of ATP oscillations were analyzed by using CE-TOF-MS analysis system. When P_ACTIN_-PxRe oscillations revealed peak or trough as shown in Figure 2, the metabolites were extracted from the ATDC5 cells. CE-TOF-MS was used for interrogating the relative levels of metabolites between peaks and troughs in P_ACTIN_-PxRe oscillations. At molecular weights range from 50.0 to 1000 *m*/*z*, 93 cationic and 109 anionic compounds were detected and analyzed in this analysis. To delineate the metabolomic alterations between peak and trough in ATP oscillations, the ratio of metabolite concentrations between peak and trough of P_ACTIN_-PxRe oscillations was represented in heatmap (Figure 3). Among total compounds, 15 cationic and 18 anionic compounds revealed statistically significant change between peak and trough of P_ACTIN_-PxRe oscillations. 

### 3.3. Metabolites to Significantly Increase at Peak of ATP Oscillations, in Comparison with That at Trough of ATP Oscillations

ATP showed higher concentration at the peak of P_ACTIN_-PxRe oscillations than at the trough (Table 1), which confirmed that P_ACTIN_-PxRe oscillations reflect ATP oscillations. Besides ATP, 3 cationic compounds such as O-acetylcarnitine, phosphorylcholine, and tyramine and 11 anionic compounds such as fructose 1,6-bisphosphate, malate, fumaric acid, 2-oxoglutarate,cis-aconitic acid, 1,3-bisphosphoglycerate, 2-phosphoglycerate, 3-phosphoglycerate, NADH,UDP-glucuronic acid, and glucuronic acid were significantly increased at the peak of P_ACTIN_-PxRe oscillations (Table 1). This result suggests that intracellular levels of these compounds oscillate in phase with ATP oscillations.

These compounds include glycolytic intermediates such as fructose 1,6-bisphosphate, 3-phosphoglyceric acid, and 2-phosphoglyceric acid and intermediates of the tricarboxylic acid cycle (TCA cycle) such as cis-aconitic acid, 2-oxoglutaric acid, fumaric acid, and malate. This result implicates that glycolysis and TCA cycle oscillate during ATP oscillations. Previous studies reported the similar results that glycolytic oscillations are coupled to oscillations in mitochondrial membrane potentials in *S. cerevisiae* and pancreatic b-cells [17–19] and suggested that glycolytic oscillations drive mitochondrial oscillations [20]. However, the present result that either a glycolysis inhibitor or a mitochondrial inhibitor prevented ATP oscillations implies that glycolytic and mitochondrial oscillations are interdependent on each other. In addition, NADH was significantly increased in the peak of ATP oscillations, indicating that NADH oscillates in phase with ATP oscillations. Although it is uncertain how NADH level stems from glycolysis or mitochondrial respiration, NADH oscillations support that glycolysis or mitochondrial respiration oscillates during ATP oscillations in chondrogenesis. 

O-acetylcarnitine level was also higher in the peak of ATP oscillations, which indicates that O-acetylcarnitine level oscillates in phase with ATP oscillations. L-carnitine and acetyl-CoA are converted to O-acetylcarnitine and CoA inside mitochondria by carnitine O-acetyltransferase, and the O-acetylcarnitine is transported outside the mitochondria where it converts back to O-carnitine and acetyl-CoA [21]. It was known that cycling of acetyl-CoA through O-acetylcarnitine plays a key role in matching instantaneous acetyl-CoA supply with metabolic demand [22]. Since acetyl-CoA is required for TCA cycle in mitochondria, O-acetylcarnitine oscillations may contribute to mitochondrial oscillations by inducing oscillations of acetyl-CoA cycling. O-acetylcarnitine oscillations may be driven by oscillatory activity of carnitine O-acetyltransferase. There is evidence that suggests that carnitine O-acetyltransferase activity is necessary for the cell cycle to proceed from the G1 phase to the S phase [23]. Therefore, oscillatory activity of carnitine O-acetyltransferase may mediate prechondrogenic condensation by modulating cellular proliferation.

In addition, both uridine diphosphate (UDP)-glucuronic acid and glucuronic acid were significantly increased in the peak of ATP oscillations. UDP-glucuronic acid is the active form of glucuronic acid for the incorporation of glucuronic acid into chondroitin sulphate [24]. UDP-glucuronic acid is produced from glucose via uronic acid pathway: glucose 6-phosphate is isomerized to glucose 1-phosphate, which then reacts with uridine triphosphate (UTP) to form UDP glucose in a reaction catalyzed by UDP-glucose pyrophosphorylase, and subsequently UDP glucose is oxidized by NAD-dependent UDP-glucose dehydrogenase to yield UDP-glucuronic acid [25]. Therefore, the uronic acid pathway of glucose conversion to glucuronic acid may oscillate during ATP oscillations. Furthermore, since NADH is produced in the uronic acid pathway, the oscillatory uronic acid pathway may contribute to generating NADH oscillations during chondrogenesis. In addition, glucuronic acid and UDP-glucuronic acid are used for synthesis of glycosaminoglycans [26], and the oscillatory uronic acid pathway can drive oscillatory synthesis of proteoglycans which are major extracellular matrix (ECM) in cartilage. Our previous report demonstrated that ATP oscillations drive oscillatory secretion and proposed that ATP oscillations lead to prechondrogenic condensation by inducing oscillatory secretion of adhesion molecules and ECM involved in cell-cell adhesion [2]. Therefore, it is suggested that glucuronic acid oscillations can lead to oscillations of ECM secretion by inducing oscillatory synthesis of proteoglycan, in cooperation with oscillatory secretion activity and consequently contribute to prechondrogenic condensation during chondrogenesis. 

### 3.4. Metabolites to Significantly Increase at Trough of ATP Oscillations, in Comparison with That at Peak of ATP Oscillations

Our analysis showed that 12 cationic compounds such as diethanolamine, glycine, guanine, cyclohexylamine, glutamine, aspartic acid, 2-aminoisobutyric acid, methionine sulfoxide, proline, threonine, asparagine, and hydroxyproline and 6 anionic compounds such as phosphoribosylpyrophosphate (PPRP), ADP, dCTP, UDP, GMP, and cGMP were significantly increased in the trough of P_ACTIN_-PxRe oscillations (Table 2). This result suggests that intracellular levels of these compounds oscillate in antiphase with ATP oscillations, which is supported by the result that ADP was included in these compounds because ADP used for ATP synthesis should be in antiphase with ATP oscillations.

PPRP which is produced from glucose via either the oxidative or nonoxidative pentose phosphate pathway is a key regulator of de novo nucleotide synthesis [27]. Therefore, nucleotide synthesis may oscillate in antiphase with ATP oscillations because the result that PRPP was decreased in the peak of ATP oscillations implies that PRPP level oscillates in antiphase with ATP oscillations. This suggestion is supported by the result that for glutamine, glycine, and aspartic acid to participate in nucleotide synthesis, purines such as guanosine monophosphate (GMP), cyclic GMP (cGMP), and guanine, and pyrimidines such as UDP and cCTP showed significantly higher level in the trough of ATP oscillations. The fact that ATP is used for synthesis of PPRP and pruine/pyrimidine [28] can explain the reason why PPRP and nucleotides oscillate in antiphase to ATP. cGMP also influences Ca^2+^ dynamics in a number of different cell types [29]. Since Ca^2+^ influx was shown to mediate ATP oscillations in chondrogenesis [2, 5], cGMP oscillations may be associated with ATP oscillations via Ca^2+^ influx. Indeed, cGMP was reported to regulate Ca^2+^ influx in pancreatic acinar cells in growth factor-stimulated NIH-3T3 cells [30, 31]. On the other hand, it should be noted that the essential amino acid threonine was also decreased in the peak of ATP oscillations. It was reported that the essential amino acid-starved condition leads to decrease in PPRP level and subsequent purine synthesis mainly by decreasing the activity of nonoxidative pentose phosphate pathway [32]. This suggests that the decreased threonine level in the peak of ATP oscillations may induce the decrease in PPRP and subsequent purine level via nonoxidative pentose phosphate pathway. 

Proline, hydroxyproline, and asparagine were significantly increased in the trough of ATP oscillations. It is known that glutamine metabolism and proline metabolism are interconnected via glutamate and pyrroline-5-carboxylate [33] and asparagine are biosynthetically derived from glutamine and aspartic acid [34]. Hydroxyproline is produced by hydroxylation of proline. Therefore, the increase in glutamine and aspartic acid in the trough may lead to the increase in proline, hydroxyproline and asparagine. It was reported that hydroxyproline is a major component of collagen which occupies a large amount of extracellular matrix in cartilage [35], and hydroxyproline and proline play key roles for the collagen stability [36, 37]. These facts indicate that oscillations in hydroxyproline and proline play a crucial role in cartilage development by modulating expression level or structure of collagen during chondrogenesis. 

**Figure 4:**
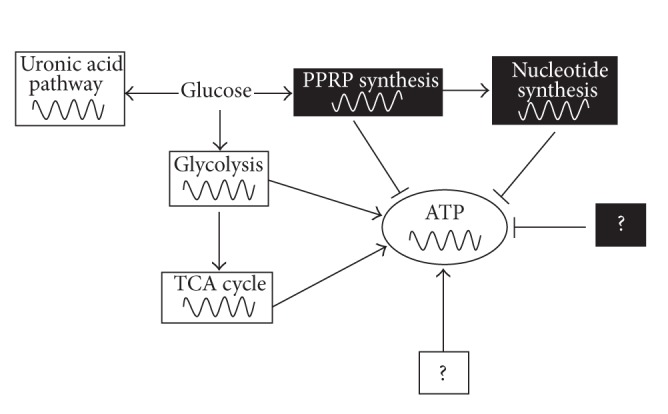
Schematic representation shows that the synchronized antiphase oscillations of the oscillatory ATP-producing metabolic pathways (glycolysis and mitochondrial respiration) and ATP-consuming pathways (PPRP and nucleotide synthesis) play a key role in generation or maintenance of ATP oscillations during chondrogenesis. White and black boxes represent oscillatory metabolic pathways in phase with ATP and in antiphase with ATP, respectively. Question marks indicate the unknown factors which are involved in ATP oscillations.

 Taken together, our results implicate that glycolysis, mitochondrial respiration, and uronic acid pathway oscillate in phase with ATP oscillations, while PPRP and nucleotides synthesis pathways oscillate in antiphase with ATP oscillations during chondrogenesis. This suggests that the ATP-producing pathways such as glycolysis and mitochondrial respiration oscillate in antiphase with the ATP-consuming PPRP/nucleotide synthesis pathway. Therefore, we propose that the antiphase synchronization of the ATP-producing and the ATP-consuming pathways can contribute to ATP oscillations during chondrogenesis (Figure 4). However, our results cannot exclude the possibility that many other factors can be involved in ATP oscillations. 

## 4. Conclusion

 Although a number of previous studies on chondrogenesis have focused on regulation at gene expression level, we recently found that metabolic oscillations play a key role in prechondrogenic condensation during chondrogenesis. The present study analyzed the quantitative change of the intermediates and products of metabolism between peak and trough of ATP oscillation in chondrogenesis and suggested that the catabolic processes such as glycolysis and mitochondrial respiration and the biosynthetic processes such as PPRP/nucleotide synthesis pathway oscillate in antiphase with each other and thus might mediate ATP oscillations during chondrogenesis. Further study is necessary to elucidate what underlies oscillations of the biochemical reactions and their integration and how the integrated biochemical reactions contribute to cartilage development, in cooperation with ATP oscillations. 

## Figures and Tables

**Table 1:** The fold changes in concentrations of metabolites increased significantly in peak of ATP oscillations.

Metabolite	Ratio (peak/trough)	*P* value (Welch's *t*-test)
Fructose 1,6-bisphosphate	1.77	0.045
Malate	1.65	0.042
Fumaric acid	1.58	0.030
2-Oxoglutarate	1.57	0.047
1,3-Bisphosphoglycerate	1.52	0.041
*cis*-Aconitic acid	1.51	0.037
2-Phosphoglycerate	1.44	0.042
3-Phosphoglycerate	1.44	0.047
NADH	1.43	0.048
UDP-glucuronic acid	1.34	0.026
*O*-Acetylcarnitine	1.32	0.011
ATP	1.31	0.042
Phosphorylcholine	1.30	0.029
Glucuronic acid	1.16	0.023
Tyramine	1.11	0.049

**Table 2:** The fold changes in concentrations of metabolites decreased significantly in peak of ATP oscillations.

Metabolite	Ratio (peak/trough)	*P* value (Welch's *t*-test)
Diethanolamine	0.88	0.020
PRPP	0.86	0.005
ADP	0.85	0.036
Glycine	0.81	0.042
Guanine	0.80	0.016
Cyclohexylamine	0.79	0.025
dCTP	0.79	0.044
Glutamine	0.78	0.014
Aspartic acid	0.77	0.032
UDP	0.76	0.045
2-Aminoisobutyric acid	0.74	0.034
Methionine sulfoxide	0.69	0.009
GMP	0.69	0.043
Proline	0.67	0.011
Threonine	0.67	0.022
Asparagine	0.64	0.032
Hydroxyproline	0.63	0.042
cGMP	0.59	0.037
